# Evidence-related requirements in Swedish public sector procurement of health and welfare technologies – a systematic review

**DOI:** 10.1186/s12913-022-07723-x

**Published:** 2022-03-17

**Authors:** Matt X. Richardson, Sara Landerdahl Stridsberg, Sarah Wamala Andersson

**Affiliations:** 1 School of Health and Welfare, Mälardalen University, Västerås, Sweden; 2 School of Health and Welfare and the Institutional Library, Mälardalen University, Västerås, Sweden

**Keywords:** Health and welfare technology, Public procurement, Evidence, Sweden

## Abstract

**Background:**

Health and welfare technologies (HWT) are becoming increasingly employed in the Nordic countries, and in Sweden in particular. The amount of HWT public procurement is likely increasing at a similar rate, but requirements for evidence for effectiveness placed on bidders during this process may be lacking.

**Method:**

This study investigated the use of evidence as a requirement in public sector tendering process of HWT, and how it affected bidder attributes and procurement outcomes. A novel type of systematic review and content analysis of requests for tenders for HWT announced prior to June 2021 was therefore conducted in Swedish public procurement databases.

**Result:**

Ninety requests for tenders for 11 types of HWT met the inclusion criteria for review, accounting for potential contracts worth 246 to 296 million EUR. Criteria requiring evidence for effectiveness were used in 16 requests for tenders, accounting for 183 million EUR in potential contracts. Eight of the requests referred to an established independent standard to confirm such evidence, such as CE standard of conformity, MDR and/or MDD. This prevalence appears to cut across all types of procuring organisations and all types of HWT. The use of any evidence criteria, or lack thereof, does not appear to affect the outcomes of the tendering process.

**Conclusion:**

Criteria requiring evidence for effectiveness are used in less than a fifth of all public procurements of health- and welfare technologies in Sweden, and less than 10% refer to some form of independent standard as confirmation of such evidence. The procurement process therefore risks creating a legacy of sub-optimal technologies in health- and social care services. More prevalent and specific requirements for evidence and its continual generation in the procurement process are highly recommended. Recommendations for decision makers, procurement managers, and developers are provided.

## Background

Health and welfare technologies (HWTs) are technology-based interventions that aim at maintaining or promoting health, wellbeing, quality of life and/or increasing efficiency in the operational delivery of welfare, social and health care services, while improving working conditions of the staff [1]. These interventions include tools, services and work methods applied to various needs and tasks of both care receivers and care providers (see Table **1**). The goal of most HWTs is to promote self-management, self-care, and independence in relation to traditional care services, including reducing the need for in-patient or institutionalized care.

**Table 1:**
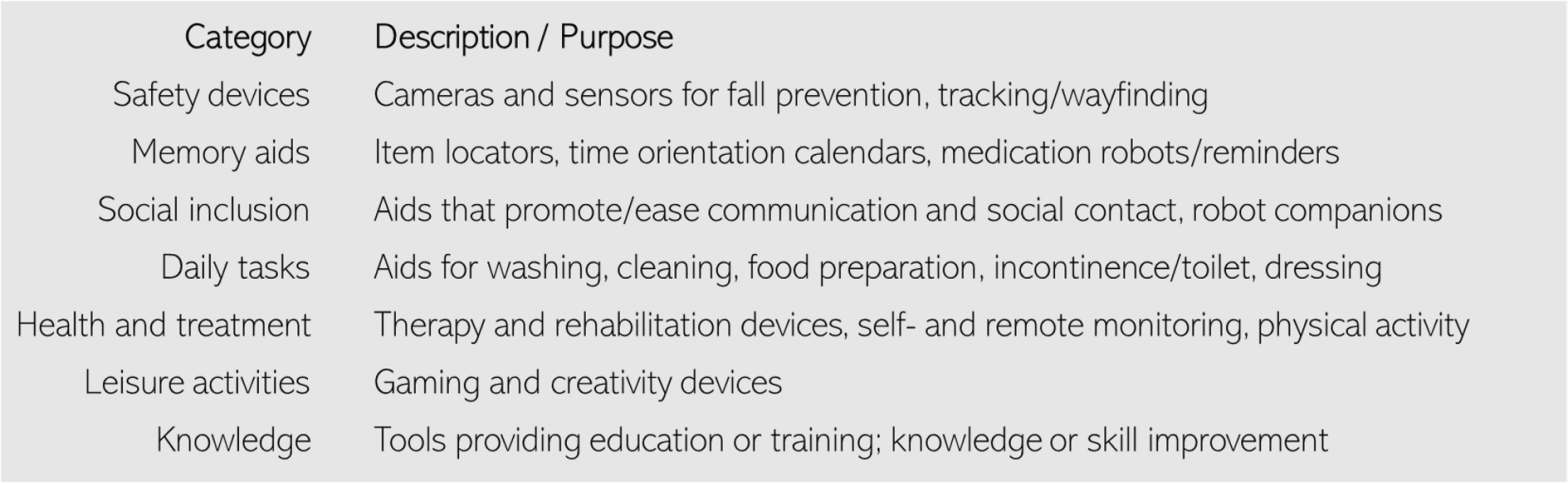
Some categories of HWT and their applications.

In Sweden, the current national vision for healthcare and social services [2] is that by 2025, the country will be world leading in eHealth and digitalization in promoting equal and effective services. Local and regional authorities that provide these services have in turn received more resources from the national level, and developed action plans to increase implementation of HWT. During 2010-2012, the Swedish government provided The Swedish Association of Local and Regional Authorities (SALAR) with 7.9 million EUR for development of e-health and welfare services in municipalities, increasing to 12.7 million EUR per year in 2013 and 2014 [3]. In 2018 this again increased to 34.5 million EUR [4], and in 2020 an additional 16.6 million EUR was provided to increase digitalization efforts in elderly care [5]. This has resulted in an exponential increase in applications, geographical scope, and number of users in both Sweden [6] and the Nordic region [7].

Logically, the public procurement of HWT has likely increased at a similar rate. Swedish procurement legislation is, as with other European Union (EU) member countries, based on EU directives (2014/24/EU being the most relevant for HWT) and EU primary law. All public bodies, including municipalities, regions, and national agencies, must follow this legislation to ensure that EU internal market competition as well as the free movement of goods and services are uninhibited, and that environmental and social considerations are taken into account. The principles of transparency, equal treatment, non-discrimination, proportionality, and mutual recognition must be followed during the procurement process. The Swedish National Agency for Public Procurement and the Swedish Competition Authority are agencies that nationally support and supervise procurement, respectively. However, neither these agencies nor any other independent organisation systematically compile the estimated amount of HWT purchased in Sweden or its outcomes. This may be due to the absence of a common definition, nomenclature, and Common Procurement Vocabulary (CPV) codes for such technologies within procurement administration.

SALAR has prioritized the implementation of four HWTs in particular: Global Positioning System (GPS)-based alarms, digital nocturnal surveillance, digital locks, and automated digital drug dispensers [8]. While HWT can potentially have positive effects on the health and wellbeing of users and the working conditions for care providers, recent systematic reviews of digital nocturnal surveillance [9] and GPS-based alarms [10] found that high-quality evidence of many proposed effects is lacking. Examples of such evidence include controlled studies, systematic follow-up and evaluation of pre-defined health, well-being, or efficiency in operational care delivery outcomes in already implemented HWT, and cost-benefit analyses among others. Many HWTs purchased for Swedish public services do not achieve expected/acceptable levels of value, either [11]. The paucity of such evidence is contradictory to the Evidence-Based Practice approach that is rigorously promoted in Swedish health and social care services as essential for decision-making processes for care and policy choices [12].

While the lack of evidence for desirable HWT effects may conceivably result from suboptimal implementation and/or use, there are upstream factors that may also play a significant role. HWT are most often purchased through an open public procurement process that is regulated by law [13] and that emphasizes the importance of value for money [14]. The European Medical Devices Regulation (MDR [15];) also requires both existing evidence for effectiveness as well as generation of new evidence during the intervention’s use, and stratifies these requirements based on the potential risk of the product. Many HWT fall under the regulation of MDR. Despite this, the contracting authority has substantial freedom in choosing what assessment criteria to use and assess [16]. As the procurement process begins upstream to implementation, it may affect evaluation and generation of evidence based on how criteria for assessing HWT are defined during purchasing.

Comprehension of the concept of evidence and its related terminology is broad in academic, social care, health, and medical professions, but in public procurement administration it may be less understood. While health- and medical specialists’ knowledge of evidence may be involved in forming the desired specifications for HWT to be procured, the translation of such knowledge into administrative requirements in legally binding documentation is uncertain.

This study investigated the use of evidence as a requirement in public sector tendering process of health and welfare technologies, with the following questions:

- What types of evidence for effectiveness and value do Swedish public sector authorities require from bidders when procuring health and welfare technologies?
- Are their differences in bidder attributes and procurement outcomes for those that require evidence for effectiveness and value compared to those that do not?

To conduct the study, a novel type of systematic review and content analysis of requests for tenders for HWT in public procurement databases was used. Systematic reviews are exhaustive and reproducible searches of existing studies or background material in relation to a research question to summarize their findings quantitatively or qualitatively. Common in the health and medical fields, such reviews can also be used in other areas such as policy or methodology. This study nonetheless appears to be the first to apply such review methodology in the analysis of publicly procured health-related technologies using a procurement database.

## Methods

### Inclusion criteria

The included population in the study were all public sector authorities within the 290 municipalities and 21 regions in Sweden or their owned subsidiaries, as well as their representative member association (SALAR) or its owned subsidiaries. Requests for tenders that were included had to involve HWT that was in accordance with the definition stated in the Background section [1]. The included requests for tenders were those that proceeded from announcement to procurement decision (regardless of appeal) at any time prior to May 2021 and had documentation in Swedish or English languages.

### Exclusion criteria

Pharmaceutical- or medical technologies used for in-patient or hospital-care settings (e.g., in-hospital digital monitoring equipment) and technologies that did not have the health or welfare of individual or organisational users or providers as a primary function (e.g., smartphones, video-conferencing systems) were excluded. Administrative systems such as electronic health records and databases/registries, interoperability applications and infrastructure, identification systems and similar were also excluded. Pre-announcements, dialogues, and requests for information were excluded, as were requests for tenders that were interrupted or cancelled by the procuring organisation prior to completion. Requests for tenders where documentation was not available were also excluded, as were duplicates found in the same or separate databases.

### Search strategy

The Mercell Opic procurement announcement database (www.opic.com [17]) was the primary database searched between May and June 2021. This database was at the time of the searches considered to be the most complete and most widely used procurement database for such purposes. Complementary searches were nonetheless conducted during the same period in the procurement announcement databases www.e-Avrop.com, www.KommersAnnons.se, and www.offentligaupphandlingar.se to check if additional HWT tenders could be found. All databases were operated by private sector companies.

### Search strings

Searches were conducted in Swedish and English. The following search words and combinations were used to identify HWT-related requests for tenders:

- English: welfare tech*; digital health tech*; digital health*; e-health*, safety alarm*; GPS-alarm*; security camera*; night camera*; nocturnal camera*; digital nocturnal surveillance; self-monitor*; remote monitor*; door alarm*; floor alarm*; absence alarm*; digital care; remote meeting*; digital lock*; robot*; incontinence sensor*; medication reminder*; medication alarm*; medication dispenser*; digital game*; digital rehab*; digital training*; smart*; health app*; digital activation*; epilepsy alarm*; seizure alarm*; digital learning*; digital education*; digital competency*;
- Swedish: välfärdsteknik*; digital hälsoteknik*; digital hälsa; e-hälsa; trygghetslarm; GPS-larm; tillsynskamera; nattkamera; digital nattillsyn; egenmonitorering; fjärrmonitorering; larmmattor; dörrlarm; rörelselarm; avvikelselarm*; digital vård*; distansmöte*; digital* lås; robot*; inkontinens sensor*; medicinpåminnare*; läkemedelspåminnare*; läkemedel dispens*medicinlarm*; läkemedelslarm*; läkemedelsautomat*; läkemedelsdispenser*; digitalt spel*; digital rehab*; digital träning*; smarta*; hälsoapp*; digital aktivering*; epilepsilarm*; digital *lärning; digital *läromedel; digital kompetensutveckling

The search words were in some cases adjusted (through addition or removal of hyphenation and spaces between words) such that several overlapping search terms were included.

### Request for tender selection process

Two researchers (MXR and SLS), hereafter referred to as reviewers, conducted the review process which had five steps:

#### Announcement screening

The request for tender announcement titles, summaries and in some cases attached documents of the obtained records were screened for relevance by one reviewer (MXR) with random checks by the other reviewer (SLS). If the reviewer(s) voted that the record fulfilled the inclusion criteria, then the record was saved in the procurement database and sorted by the search term used to identify it. Any conflicts were resolved through dialogue until consensus was achieved.

#### Request for tender documentation screening

For requests proceeding to this step, all tendering documents related the request for tender announcement were downloaded and screened for confirmation of inclusion by one reviewer (MXR) with random checks by the other reviewer (SLS). If the reviewer(s) voted that the request for tender still fulfilled the inclusion criteria, then the documentation was compiled and proceeded to data extraction. Any conflicts were resolved through dialogue until consensus was achieved.

**Fig. 1:**
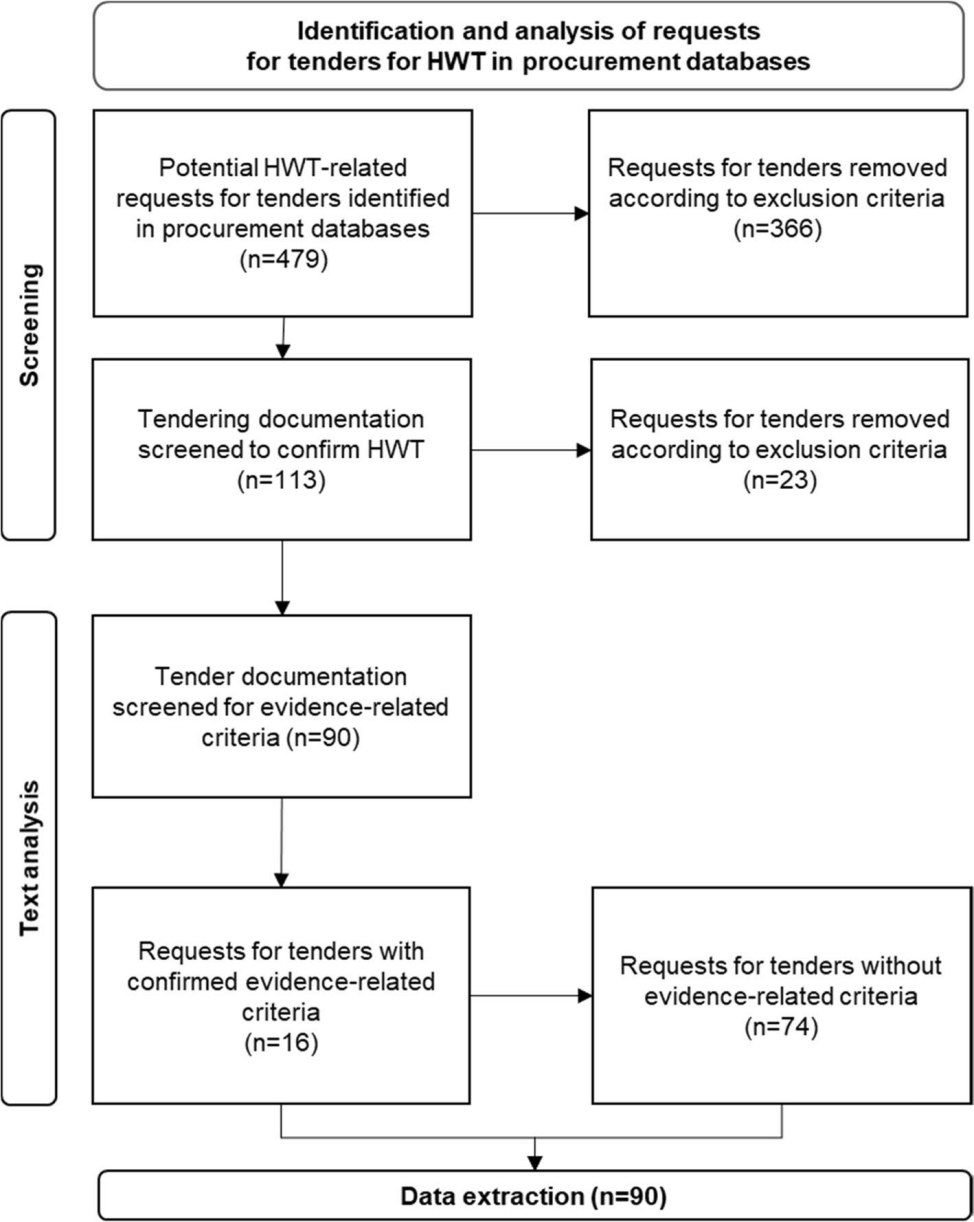
Flowchart of the search and inclusion process for HWT requests for tenders

A summary of the results of this selection process can be found in Fig. 1**.**

#### Content screening for evidence-related terminology

The textual content of all documents for included requests for tenders, including eventual awarding decisions and documentation, were then compiled in a text analysis tool (www.voyant-tools.org [18];) and searched for evidence-related terminology. The terminology searches were conducted in the same language as the included tendering documentation. The following search words and combinations were used:

English: evidence; science; scientific; sensitivity; specificity; study; studies; intervention*; benefit*; effectiveness; effect*; certification*; MDR; CE conform*; MDD; 2017/745.

Swedish: Evidens*; vetenskap*; känslighet*; specificitet*; studie*; utfall*; intervention*; *nytta; nytto*; effekt*; certifier*; bevis*, MDR; CE-märk*; MDD; 2017/745.

A context for all found terms, consisting of the 15 words to the left and right of identified search term, were copied and exported to a spreadsheet.

#### Content confirmation for evidence-related criteria

The contexts from the content screening for evidence-related terminology were then reviewed in the tendering documentation from which they were extracted by one reviewer (MXR) with random checks by the other reviewer (SLS). The context was included as an evidence-related criterion if it met the following inclusion criteria:

- Clear evaluation- or awarding criteria (in Swedish respectively known as *utvärderingskriterier* or *tilldelningskriterier*, often in the form of “shall” or “should” requirements) were used in the tendering documentation regarding evidence for effects or value, consisting of collected or objective data, a certification that is only achieved upon provision of such data, or similar *and*
- the requirement was specific to the HWT intervention in the actual request for tender.

The context was excluded as a requirement if any of the following exclusion criteria were met:

- the requirement was solely related to the bidder’s overarching organisation (e.g. that the organisation is ISO-certified or uses a quality management system); *or*
- the requirement is solely related to administration of the intervention (e.g. availability, delivery, user education); *or*
- the requirement did not address a defined outcome (e.g. “improve effectiveness” without stating what that effectiveness involves or means for the organisation); *or*
- there was no request for documentation, certification or data to demonstrate fulfilment of the requirement (e.g. a simple “Yes/No” response sufficed as fulfilling the requirement).

The criteria confirmation was conducted by one reviewer (MXR), with random checks by the other reviewer (SLS). Any conflicts were resolved through dialogue until consensus was achieved.

#### Data extraction

Essential information regarding the type of HWT, procuring organisation, duration of the tender, number of bidders and related information was extracted for all included requests for tenders. The use of evidence criteria was also denoted with a description of what manner it was used (e.g., as a qualifier for bidding, for ranking of bids, etc.). If the tender was successfully awarded, then the economic value of the tender and the attributes of the winning bidder were also extracted; if the tender was appealed then this was also noted, along with information regarding the outcome of the appeal, if available.

### Outcomes of interest

The main outcomes of interest for the analysis were:

- the prevalence and type of terminology in the tendering documentation related to evidence for effects and/or value
- the prevalence and type of evidence-related criteria when evaluating bids
- the attributes of the procuring organisation, bidders, type of HWT and procurement result in relation to the above outcomes

## Results

There were 343,343 requests for tenders available in the main procurement database that was searched. No tenders were found in additional procurement databases that could not be found in the main database.

After applying the HWT-related search terms, 479 requests for tenders were identified that could include HWT (Fig. 1). Of these, 389 were removed during the steps in the screening phase for not meeting the inclusion criteria and/or according to one or more of the exclusion criteria. Of these excluded requests for tenders, eighteen were excluded solely because they were cancelled by the procuring organisation during the tendering process.

Ninety requests for tenders announced between January 2008 and May 2021 progressed to the text analysis stage, containing 11 different types of HWT (Table 2). Of these, digital and GPS-based safety alarms were the most requested (34 requests), followed by automated pharmaceutical dispensers/robots (17 requests) and digital locks or entry systems (10 requests). Medium and small municipalities had the most requests, at 37 and 27, respectively. The total value for the 90 tenders was at least 246 million EUR.

**Table 2:** Description of HWT procurements included in the text analysis stage. The number of requests for tender announced by the procuring organisation for specific types of HWT are in parentheses

Type of procuring organisation	HWT type	Use of evidence-related criteria
Small municipalities (27 requests for tenders)	Automated pharmaceutical dispensers/robots (4) Combined HWT systems/packages (3) Digital locks/entry systems (3) Digital/GPS safety alarm with or without response chain (17)	14.8% (4)
Medium municipalities (37 requests for tenders)	Automated pharmaceutical dispensers/robots (7) Combined HWT systems/packages (6) Digital cognitive support (1) Digital locks/entry systems (4) Digital rehabilitation aids (1) Digital/GPS safety alarm with or without response chain (12) Digital surveillance (4) Digital therapy animals/robots (1) Digital training aid (1)	16.24% (6)
Large municipalities (2 requests for tenders)	Digital locks/entry systems (1) Digital/GPS safety alarm with or without response chain (1)	0
Small regions (2 requests for tenders)	Digital cognitive support (1) Digital surveillance (1)	0
Medium regions (5 requests for tenders)	Automated pharmaceutical dispensers/robots (1) Digital epilepsy/seizure alarm (1) Digital surveillance (1) Self-monitoring technologies (1)	40% (2)
Large regions (4 requests for tenders)	Automated pharmaceutical dispensers/robots (2) Combined HWT systems/packages (1) Digital/GPS safety alarm with or without response chain (1)	50% (2)
Other^a^ (13 requests for tenders)	Automated pharmaceutical dispensers/robots (3) Digital locks/entry systems (2) Digital/GPS safety alarm with or without response chain (5) Digital surveillance (2) Self-monitoring technologies (1)	14.2% (2)

^a^ Includes consortiums of municipalities and/or regions, and municipality- or region-owned companies

The requests for tenders that underwent text analysis contained in total 983 documents. 258 contextual phrases containing evidence-related terminology were identified after screening these documents. After full-text contextual screening, 93 phrases were identified as evidence-related criteria in the request for tender. These criteria were found in 16 unique requests for tenders (Table 3).

**Table 3:** Summary of characteristics for HWT requests for tenders included in the review that used evidence-related criteria. The evidence-related search term found in the text analysis is highlighted in bold text

HWT type and year of request	Type of procuring organisation^**a**^ and method	Tender value, EUR (max. Contract length)	Procurement outcome^**b**^ (number of bidders)	Evidence-related criteria
Digital/GPS safety alarm, 2021	Small municipality, open procurement	€155,000 (8 years)	Awarded to 1 SME (3 bidders)	A system description must be provided as **proof** that the requested functions can be delivered; this will be assessed by an evaluation group.
Digital/GPS safety alarm, 2021	Small municipality, open procurement	€1,133,950 (8 years)	Awarded to 1 SME (4 bidders)	A system description must be provided as **proof** that the requested functions can be delivered; this will be assessed by an evaluation group.
Self-monitoring technologies, 2021	Medium region, simplified procurement	not available (10 years)	Appealed; Awarded to 1 MNC (bidders not available)	Medical personal will assess if the monitoring solution described is in line with scientific **studies** and national guidelines.
Self-monitoring technologies, 2021	Large region, negotiated procurement	€15,342,800 (7 years)	Ongoing	The provider’s solution must meet **MDR 2017/745 CE standard of conformity** alternatively **MDD 93/42** during the entire contract period. The user must be able to receive **evidence-based**, or alternatively region-formulated, self-care guidance to improve treatment.
Automated pharmaceutical dispensers/robots, 2020	Medium municipality, simplified procurement	€295,800 (4 years)	Awarded to 1 SME (1 bidder)	The bidder must provide a **CE standard of conformity** document, a clinical **study** (brief report) and a clinical test report to show that the requirements placed on the product/service are met
Self-monitoring technologies, 2020	Medium region, open procurement	€1,045,100 (4.5 years)	Awarded to 1 MNC (6 bidders)	Bidders must be able to provide a **certificate** showing that the product meets the **CE standard of conformity** and **MDR 2017-745** for those aspects that involve medical technical products.
Digital locks/entry systems, 2020	Medium municipality, open procurement	€315,700 (7 years)	Appealed; Awarded to 1 SME (3 bidders)	Product must meet industry security standard for class S3 locks and provide proof of **certification** from a nationally accredited third-party **certification** institute
Digital/GPS safety alarm, 2020	Medium municipality, open procurement	€2,395,900 (8 years)	Awarded to 2 SME (5 bidders)	**Proof** that the alarm meets a standard IP-classification; documentation confirming this is required.
Digital/GPS safety alarm, 2020	Member-owned corporation, open procurement	€158,739,550 (4 years)	Ongoing	**Proof** that the alarm to be worn does not contain specific chemicals; a signed assurance from the manufacturer, a chemical analysis protocol, or a full declaration of contents must be provided. **Proof** that the alarm communicates via an open protocol that meets a technical standard; for example, a test protocol from an external testing institute can be provided.
Digital/GPS safety alarm, 2020	Medium municipality, simplified procurement	€31,335 (13 years)	Awarded to 1 SME (2 bidders)	Products that fall under the regulations for **CE standard of conformity** must meet the requirements stated in the actual **CE standard of conformity**. Documentation that shows that these requirements are met must be available upon request.
Digital locks/entry systems, 2020	Medium municipality; Regionally owned corporation	€ 104,700 (4 years)	Awarded to 1 SME (1 bidder)	The lock solution and the key safe must be approved and **certified** by the national association according to standard SS3522 / SSFN-024 class 3 or higher, or a similar **certificate**. The **certificate** must be included in the bid.
Digital/GPS safety alarm, 2019	Small municipality, open procurement	€ 1,577,600 (4 years)	Appealed, no winner (3 bidders)	Regarding communication frequencies and standards, the equipment that falls under the directive for the **CE standard of conformity** must at the time of delivery meet the requirements for **CE standard of conformity**
Automated pharmaceutical dispensers/robots, 2019	Small municipality, open procurement	€ 399,650 (4 years)	Awarded to 1 SME (2 bidders)	All products shall meet the **CE standard of conformity**, the national law on medical technical products (SFS 1993:584) and the national standard SS-EN 980
Automated pharmaceutical dispensers/robots, 2018	Medium municipality, direct procurement	not available (0.5 years)	Awarded to 1 SME (1 bidder)	Requirement that the product should have test documentation and a valid **CE standard of conformity**; bidder should describe how they meet these requirements.
Digital locks/entry systems, 2018	Medium municipality, open procurement	€ 957,800 (4 years)	Awarded to 1 SME (1 bidder)	Product must meet industry security standard for class 3 locks, and proof of **certification** must be provided in the bid
Digital/GPS safety alarm, 2017	Large region, open procurement	€973,200 (4 years)	Awarded to 3 SME (4 bidders)	Products must meet **CE-standard of conformity** according to **MDD**

^a^ Small municipality: under 30,000 inhabitants; Medium municipality: between 30,000 and 250,000 inhabitants; Large municipality: over 250,000 inhabitants; Small region: under 250,000 inhabitants; medium region: between 250,000 and 1,000,000 inhabitants; large region: over 1,000,000 inhabitants

^b^ *SME* Small or medium-sized enterprise,  *MNC *Multi-national corporation

Tenders for four different types of HWT were requested in the 16 announcements with a total potential value of 183,492,600 EUR: digital/GPS-based safety alarms [7], self-monitoring technologies [3], automated pharmaceutical dispensers/robots [3], and digital locks/entry systems [3] (Table 3). Medium municipalities had the most requests [6], followed by small municipalities [4], other consortiums and organisations [4] and medium regions [2]. Thirteen tenders were successfully awarded including in two cases of appealed decisions, two were still ongoing at the time of publication, and one was appealed and as a result not awarded. The evidence-related criteria most used were requiring a CE standard of conformity [7], followed by some other form of proof [5], certification [3], scientific or clinical studies [2]. Three requests required that the product or service fulfilled the requirements placed by the MDR or MDD, alongside requirements for the CE standard.

In comparing the requests for tenders that used evidence-based criteria with the requests that did not, no differences were seen in the average number of bidders or average maximum contract length (Table 4). The number of awarded and unawarded tenders due to appeal was also the same for both; a relatively higher number of appeals in the requests for tenders using evidence-related criteria was noted, though. There were 12 unique winning bidders across the requests for tenders that used evidence-based criteria; two small or medium-sized enterprises (SME) won 3 separate tenders, and another SME won 2 tenders. All others won only 1 tender each.

**Table 4:** Comparison of requests for tenders using evidence-related criteria to those that did not

Use of evidence-related criteria	Average number of bidders	Procurement outcome^**a**^	Average max. Contract length	Type of procuring organisation
Yes (*n* = 14) ^**a**^	3.8	Awarded: 78.6% Awarded after appeal: 14.3% Appealed, not awarded: 7.1%	3.8 years	Small municipality: 28.5% Medium municipality: 42.8% Large municipality: 0% Small region: 0% Medium region: 7.1% Large region: 7.1% Other consortium: 14.3%
No (*n* = 70) ^**a**^	3.9	Awarded: 92.9% Appealed, not awarded: 7.1%	3.9 years	Small municipality: 32.9% Medium municipality: 44.3% Large municipality: 2.9% Small region: 2.9% Medium region: 5.7% Large region: 2.9% Other consortium: 14.3%

^**a**^ The requests for tenders that were still ongoing at the time of publication or for which no outcome was otherwise available were excluded in the n value and in the percentages in Procurement outcome and Type of procuring organisation

## Discussion

Criteria requiring evidence for effectiveness are used in less than a fifth of all public procurements of health- and welfare technologies in Sweden. Less than 10% of all HWT procurements refer to some form of independent standard to confirm such evidence. This prevalence appears to cut across all types of procuring organisations and all types of HWT. The use of evidence criteria, or lack thereof, does not appear to affect the outcomes of the tendering process, yet there appears to be a diversity of bidders of different sizes that win tenders.

In this review, the 90 identified HWT tenders potentially accounted for 246 to 296 million EUR of public funds if successfully contracted, of which 183 million EUR were allocated to tenders that used evidence-related criteria. Almost 87% of this amount, or 158.7 million EUR, was accounted for by a single procurement of digital safety alarms by a local and regional authority member organisation, however. The average HWT contract length was almost four years, with many tenders lasting 8-10 years. A lack of requirement of evidence for effectiveness implies that a legacy of sub-optimal technologies in health- and social care services may be created during the procurement process and be established as the norm. The lack of verifiable effects found in recent reviews of nocturnal surveillance technologies [9] and GPS-based safety alarms [10] - both widely implemented HWT in the Nordic countries – show that this may already be occurring on a larger scale. Potential risks and additional resource demands on public authorities and their users that arise from not achieving beneficial health or welfare effects over the lifecycle of implemented technologies may far outweigh the initial purchasing amounts.

Reasons for the lack of use of evidence-related criteria in HWT procurement can be hypothesized from previous research. One recent Swedish study [19] elucidated several challenges in application and deployment of HWT at various stages of procurement, arising from economic resources, standardisation, and interoperability, among others. These challenges may potentially deflect the attention of decision makers from evidence-related criteria regarding effectiveness, and direct it towards more immediately assessable technical and economic aspects. The latter in particular often appears to have priority: a survey of prospective providers [20] found that more than half felt that public authorities’ requests for tenders placed too much weight on low prices, and too little weight on quality, product outcomes and follow-up of procurement. Other research has found that operational behaviour in procurement may largely be driven by the training that procurement administration personnel receive, as well as previous habits that tend to remain unscrutinised, which was referred to as “institutional inertia” [16].

The European MDR requires both existing evidence for effectiveness as well as generation of new evidence during the intervention’s use, and stratifies these requirements based on the potential risk of the product. In this manner, requiring that bidders achieve MDR compliance would be a suitable proxy for providing evidence for effectiveness. In the current review, the MDR was used as a criterion in only two requests for tenders. While the CE standard of conformity was used in seven requests for tenders (including the two that referred to the MDR), most could have been interpreted as referring to the earlier 1993 Medical Device Directive (MDD [21];) despite the MDR having been established in 2017 and recently fully implemented. This would mean that they would likely not meet the standards of the MDR, where many HWT are placed in a higher risk classification with stricter requirements regarding clinical and real-world evidence (RWE) and external auditing. Other types of studies to confirm evidence of effect were requested in only two tenders.

Even if requirement of compliance with the newly implemented MDR becomes more prevalent in public procurement, evidence requirements may still need to be taken more seriously by procuring stakeholders. The “outcomes that matter” for any HWT application need to be formed by the procuring organisation in terms that make them relevant to local conditions and preferences. The structures and processes to accumulate evidence for HWT effects – both prior to and after implementation - in such local conditions need to be spelled out in clear terms to be able to maintain the integrity of a contract with any provider. Such requirements should be based on well-defined intentions at the start of a tendering process such that the integrity of evidence-based criteria, when being translated into legally binding procurement documentation, occurs successfully and in line with the intention.

The procurement of pharmaceuticals and medical technology is, in comparison to current HWT procurement, vastly more evidence based. Scientific and statistical validity, analytical and clinical performance, and peer-review acceptance pertaining to pre-defined intended benefits are required by most continental and national authorities to achieve certification, with results from standardized clinical trials often necessary to achieve this. HWT currently lacks a similar national framework for assessment of effectiveness. Development of such a framework, with a national authority to oversee its use, may thus promote a broader use of evidence-based criteria in public procurement and minimise unintended costs and risks.

### Recommendations for stakeholders

- National and/or regional policy makers should task appropriate authorities to develop and oversee a framework for evidence based HWT use, to support public sector procurers and developers achieve a common understanding of evidence use. The MDR should be a foundation in this framework, but also provide guidance adjustable to local conditions and user preferences.
- Decision makers that initiate procurement processes should clearly define intentions and outcomes that matter – such as health, welfare, and efficiency in operational care delivery outcomes - for any HWT application, and in terms of the local conditions and known or expected user preferences. These intentions and outcomes will form the core of what pre-existing evidence is necessary, and what evidence needs to be generated during the lifecycle of the HWT.
- Procurement officials must establish an evidence-based procurement process based on the intentions and outcomes prioritized by decision makers. Input should be obtained from researchers, developers and users when establishing this process. The resulting process should include a list of adaptable criteria for what evidence must be provided by bidders and how, as well as criteria for real world evidence generation after purchase and implementation. Evaluation of bids should also place significant weight on assessing such evidence, and contracts with winning bidders should include clauses regarding continual evidence generation as a condition for contract fidelity and/or extension. The use of MDR as a standard criterion should be vastly increased.
- For HWT developers, evidence for application effectiveness as well as real world evidence generation after implementation are now engrained in the EU MDR. High-quality continual evidence generation should be planned for when developing applications and be adaptable to expected purchasers’ local conditions and needs and user preferences. For small- and medium-sized enterprises, partnership with both academia and presumptive purchasers and their users to assist in meaningful data collection and evidence summarization may be necessary in many cases.
- Academia can play a significant role in creating a neutral place where stakeholders on the demand and supply sides can meet for mutual learning, sharing and co-creation of both innovations and evidence generation for HWT.

## Conclusion

The market for health and welfare technology is growing rapidly, particularly in Sweden. At the same time, evidence requirements during public procurement of HWT in Sweden occur in only a small portion of all requests for tenders, which may result in a legacy of ineffective applications, risks, and excess costs. Policy makers and procurement officials should take steps to establish transparent and rigorous processes for evidence requirement and assessment with the MDR as a starting point. These stakeholders should also, together with HWT developers and researchers, work together to achieve high-quality, locally relevant evidence generation when purchasing and employing HWT.

## Data Availability

The datasets used and/or analysed during the current study are available from the corresponding author on reasonable request.

## Abbreviations

CE: Conformitè Europëenne
CPV: common procurement vocabulary
EU: European Union
GPS: Global Positioning System
HWT: health and welfare technology
MDD: European Medical Devices Directive
MDR: European Medical Devices Regulation
MNC: Multinational Corporation
SALAR: Swedish Association of Local and Regional Authorities
SME: Small- or medium-sized enterprise
